# M Mode Ultrasound and Tissue Doppler Imaging to Assess Diaphragm Feature in Late Onset Pompe Disease

**DOI:** 10.3390/neurolint12030012

**Published:** 2020-11-13

**Authors:** Paris Meng, Adam Ogna, Abdallah Fayssoil

**Affiliations:** 1 Intensive Care Unit and Home Mechanical Ventilation Unit, Raymond Poincaré Hospital, APHP, 92380 Garches, France; parismeng@ymail.com (P.M.); adam.ogna@gmail.com (A.O.); 2 Cardiology Unit, Paris Saclay University, Raymond Poincaré Hospital, APHP, 92380 Garches, France

**Keywords:** LOPD, diaphragm, ultrasound

## Abstract

Late-onset Pompe disease (LOPD) is an autosomal recessive lysosomal storage disease. Clinical features include skeletal muscle deficiency and diaphragm weakness. Clinical management relies on supportive treatment and mechanical ventilation in patients with chronic respiratory failure. M mode ultrasound and sniff tissue Doppler imaging can be used to assess and follow diaphragm function.

## 1. Introduction

Late-onset Pompe disease (LOPD) is an autosomal recessive lysosomal storage disease. This disease is related to a defect in the activity of the glycogen degrading lysosomal enzyme, the alpha 1–4 glucosidase enzyme (GAA), causing glycogen accumulation and muscle weakness [1]. Clinical features include skeletal muscle deficiency and diaphragm weakness. Clinical management relies on supportive treatment that includes enzyme replacement therapy and mechanical ventilation. Respiratory muscle monitoring is essential in this disease, since respiratory insufficiency and sleep disordered breathing are frequent in patients with LOPD and an important cause of morbidity [2,3]. Diaphragm, the main inspiratory muscle, has a crucial role in breathing during sleep. Diaphragm weakness is often associated with sleep-disordered breathing. Recently, ultrasound has been used to assess diaphragm weakness in LOPD [4]. Here, using M mode ultrasound and tissue Doppler imaging (TDI) [5], we report diaphragm weakness attested by a paradoxical motion during a sniff test in a patient with LOPD. The study was performed in compliance with the ethical principles formulated in the declaration of Helsinki and was approved by the French regulatory board (*CNIL, commission nationale de l’informatique et des libertés*). Informed consent was obtained from the patient for this case report.

## 2. Case Report

**Figure 1:**
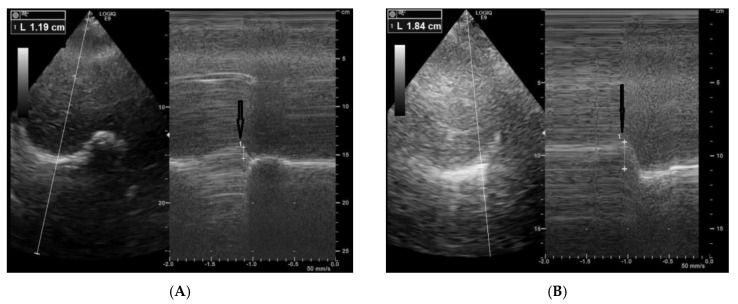
(**A**) (−11 mm) and (**B**) (−18 mm) hemi diaphragm paradoxical displacement using M mode ultrasound during a sniff maneuver in patient with LOPD.

**Figure 2:**
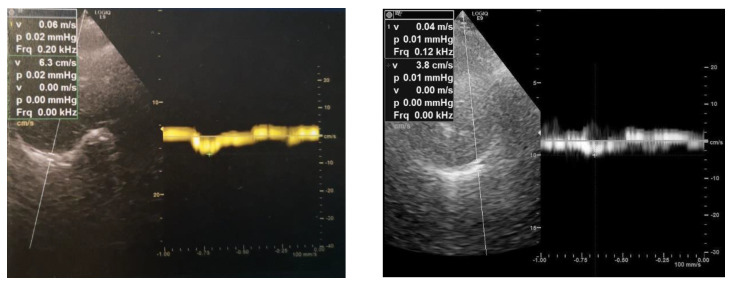
Diaphragm right peak negative velocity (−6 cm/s) and left peak negative velocity (−4 cm/s) using tissue Doppler imaging coupled to a sniff maneuver in patient with LOPD.

A 64-year-old female patient was referred to our unit for a cardiorespiratory evaluation because of dyspnea and orthopnea. She was treated with enzyme replacement therapy for 10 years because of LOPD. She had limb girdle weakness with waddling gait. The Walton score was 3. The other clinical parameters were as follows: body mass index at 26 kg/m^2^, systolic blood pressure at 109 mmHg, diastolic blood pressure at 65 mmHg, and diurnal oxygen transcutaneous saturation at 100%. Doppler Echocardiography showed a normal left ventricular ejection fraction (61%) with normal cardiac loading and subnormal systolic arterial pulmonary pressure (41 mmHg). We performed a diaphragm exploration using ultrasound in the same exam. From the subcostal view, we assessed the diaphragm motion using TM mode in rest and during a sniff maneuver, as previously described [5]. We found a paradoxical diaphragm motion during a sniff maneuver of both the right (−11 mm) and the left hemi diaphragm (−18 mm), with M mode (Figure 1). Using tissue Doppler imaging, we also found a negative and reduced peak velocity of diaphragm during the sniff maneuver, measured on the right hemi diaphragm (−6 cm/s) and on the left hemi diaphragm (−4 cm/s) (Figure 2). Diaphragm weakness was confirmed by the decrease in maximal inspiratory pressure (22 cm H_2_O), the decrease in sniff inspiratory pressure (29 cm H_2_O) and the drop in the predicted value of respiratory forced vital capacity (VC) from upright to supine position (from 43% to 27%). In the meantime, with transcutaneous capnometry, the patient disclosed nocturnal hypoventilation attested by an increase in the transcutaneous PCO_2_ (82% of the registration time with PCO_2_ > 50 mmHg) and a decrease in transcutaneous PO_2_ (39% of time with PO_2_ < 90%). Non-invasive nocturnal ventilation was introduced to manage the respiratory failure.

## 3. Discussions

We report this case, in which bedside diaphragm evaluation by ultrasound suggested diaphragm weakness with nocturnal hypoventilation as the cause of dyspnea and orthopnea, in a patient with LOPD. In LOPD, clinically relevant diaphragm weakness may develop even in patients with little peripheral muscular impairment, causing nocturnal hypoventilation, supine dyspnea aggravation, daytime hypercapnia, fatigue and excessive daytime sleepiness [3]. Diaphragm weakness is often associated with limb girdle weakness [6]. NIV has been shown to normalize gas exchange and improve respiratory status and symptoms in this context [1,7]. Respiratory involvement in LOPD can be subtle and it is essential to monitor patients with repeated respiratory function tests, measuring vital capacity, maximal inspiratory pressure, and maximal expiratory pressure. The disease can affect not only the diaphragm, but also the upper airways and the other respiratory muscles [1]. Classically, the drop in the VC from upright to supine is an indirect marker of diaphragmatic weakness [8]. To assess the inspiratory muscle strength, sniff inspiratory nasal pressure and maximal inspiratory mouth pressure can be used. Sniff maneuver coupled with ultrasound can be used to selectively assess diaphragm function [5,9]. Regular respiratory function monitoring is crucial in LOPD. In fact, LOPD patients with a supine VC < 60% of predicted value frequently have sleep-disordered breathing, and nocturnal hypoventilation is frequent when VC is below 40% [10]. M mode ultrasound and Tissue Doppler imaging may be applied in patients with LOPD to assess and monitor the diaphragm function, with the advantage of being applicable at the bedside, without the need for lung function facilities. In this case report, the paradoxical motion and the negative velocity of the diaphragm during the sniff manoeuver gave the necessary clues to further investigate respiratory function, allowing the depiction of the presence of nocturnal hypoventilation. This finding highlights the potential application of ultrasound to monitor diaphragm in LOPD. Current guidelines recommend introducing noninvasive ventilation in patients with LOPD relying on the values of blood gas exchange, MIP, FCV and sleep studies. Future studies will be necessary to assess the additive value of diaphragm ultrasound in this field.

## 4. Conclusions

In LOPD, bedside ultrasound may be used to screen for diaphragmatic dysfunction. In the presence of a diaphragm paradoxical motion or a negative TDI velocity during a sniff maneuver, nocturnal transcutaneous capnometry should be performed.
